# Risk Aversion, Organ Utilization and Changing Behavior

**DOI:** 10.3389/ti.2022.10339

**Published:** 2022-04-07

**Authors:** Adnan Sharif

**Affiliations:** ^1^ Department of Nephrology and Transplantation, University Hospitals Birmingham, Birmingham, United Kingdom; ^2^ Institute of Immunology and Immunotherapy, University of Birmingham, Birmingham, United Kingdom

**Keywords:** decision making, organ utilization, psychology, risk aversion, risk tolerance, discard

## Abstract

Improving organ acceptance and utilization rates is critical to ensure we maximize usage of donated organs as a scarce resource. Many factors underlie unnecessary discard of viable organs. Declined transplantation opportunities for candidates is associated with increased wait-list mortality. Technological advancements in organ preservation may help bridge the gap between donation and utilization, but an overlooked obstacle is the practice of risk aversion by transplant professionals when decision-making under risk. Lessons from behavioral economics, where experimental work has outlined the impact of loss or risk aversion on decision-making, have not been translated to transplantation. Many external factors can influence decision-making when accepting or utilizing organs, which are potentially amendable if external conditions are improved. However, attitudes and perceptions to risk for transplant professionals can pervade decision-making and influence behaviour. If we wish to change this behavior, then the underlying nature of decision-making under risk when accepting or utilizing organs must be studied to facilitate the design of targeted behavior change interventions to convert risk aversion to risk tolerance. To ensure optimal use of donated organs, we need more research into decision-making under risk.

## Introduction

Due to continued disparity between the supply versus demand for organs, maximizing usage of available organs is critically important. Strategies to increase both organ acceptance and utilization have been published, with the United Kingdom one example (1), acknowledging wide disparities in organ acceptance and/or utilization across national transplant programs. Some of this heterogeneity is unavoidable, relating to center-specific or cohort-specific factors, and multi-stakeholder calls to action acknowledge these barriers (2). However, another important variable is risk aversion. Specifically, risk aversion from transplant professionals when they receive viable organ offers but decision-making is skewed *against* acceptance and/or utilization. Risk aversion may occur due to infrastructural constraints, resource pressures or organ quality concerns. While the latter concern may be attenuated with development of novel techniques (e.g., normothermic perfusion), current financial realities limit the possibility of significant monetary investment into staffing and/or resources. Wide heterogeneity *between* centers can be explained by these confounders and is well documented. However, *within* center heterogeneity exists but is poorly described. Disparate practice by individuals is influenced by risk psychology, but estimating its true prevalence is difficult without internal audit and governance measures.

While this issue has not been completely overlooked in the transplant literature (3), targeted research pales in comparison to other areas. However, even with better tools like real-time risk calculators, biomarkers, artificial intelligence algorithms, etc., decision-making for some transplant professionals will still favor risk aversion over risk tolerance due to individualized cognitive biases. After summarizing the problem, I hope to argue for a proactive way forward to tackle the risk psychology component in organ offer decision-making.

### Organ Utilization Data

Many viable organs are discarded. Using kidneys as an example, Mohan et al. observed 17.3% of procured kidneys in the United States between 2000 and 2015 were discarded, with considerable geographical variation (4). Donor kidneys with multiple unfavorable characteristics were more likely to be discarded. However, some unilaterally discarded kidneys had favorable donor characteristics, evidenced by recipients of the non-discarded partner kidneys experiencing 1-year death-censored graft survival rates >90%. Exploring the last 2 decades in the United States, Stewart et al. observed >80% of kidney discard rates between 1987 and 2015 could be explained by the broadening donor pool, but the presence of unexplained residual factors suggested behavioral factors at play (5).

Organ discard rates in European countries are lower than the United States (6). If deceased donor kidney acceptance in the United States mirrored the French model (discard rate 17.9% versus 9.1%, respectively, *p* < 0.001), then Aubert et al. hypothesize 62% of discarded kidneys (*n* = 17,435) could generate 132,445 allograft life-years (7). Efforts to address this imbalance have been initiated. In the United States, new metrics for performance monitoring of transplant programs were approved in December 2021 (8). Compared to only post-transplant factors previously monitored (1-year patient/graft survival alone), new metrics include two additional post-transplant measures (90-day graft survival and 1-year graft survival conditional on 90-day graft survival) and importantly two new pre-transplant measures for each transplant program: 1) the rate of pre-transplant deaths, and; 2) the ratio of organ offers made to and accepted for candidates. These metrics are important as, while death or removal from the waiting-list is an unfortunate outcome for anyone awaiting a solid organ offer, for such a waiting list outcome to occur after refusal of a viable organ offer (i.e., accepted by another center on behalf of another wait-list candidate) is a travesty.

### Outcomes for Candidates of Declined Offers

Declined organ offers is not a benign event for wait-listed candidates. Husain et al. studied a United States cohort of 280,041 wait-listed kidney transplant candidates (9). They observed approximately 30% of candidates who received at least one deceased-donor offer that was declined on their behalf eventually died or were removed from the waiting list. Odds for death on the waiting-list varied significantly across the country. Choi et al. studied a United States cohort of 9,628 wait-listed heart transplant candidates between 2007 and 2017 (10). They observed every 10% increase in center-adjusted acceptance rate for organ offers made to the highest-priority candidates was associated with a 27% reduction in the mortality rate among patients on the waitlist, with no detriment in 5-year adjusted post-transplant patient or graft failure. Center variability was dramatic, with acceptance rates to first-rank candidates varying nationally between 12.3% and 61.5% after adjustment for donor, candidate and geographical variables. Among 19,703 unique organ offers, only 6,302 hearts (32.0%) were accepted for first-rank candidates. Similar acceptance rates are observed after liver transplantation. Goldberg et al., in another cohort study undertaken in the United States, observed 8,882 out of 23,740 unique organ offers (37.4%) were accepted for first-ranked liver transplant candidates (11). After adjustment for organ quality and burden of illness in wait-listed candidates, the adjusted center-specific organ acceptance rates varied nationally between 15.7% and 58.1% (*p* < 0.001). In multivariable models, there was 27% increased odds of waitlist mortality for every 5% absolute decrease in center-adjusted organ offer acceptance rate (adjusted Odds Ratio 1.27, 95% Confidence Interval 1.20–1.32). While there may be genuinely valid clinical reasons for declining organs for first-ranked candidates, the influence of non-clinical factors for some declines cannot be ignored.

### Lessons From Behavioral Economics

Perhaps the most difficult challenge in organ transplantation is deciding to accept or decline an offered organ. Risks associated with the donor or organ must be balanced against the survival prospects of the wait-listed candidate. Translating national statistics and population-level data to the individual for personalized decision-making is fraught with challenges. Transplant professionals will complement objective evidence with their subjective perception and experience, which can result in markedly variable assessment of risk versus benefit. If we translate classic economic theory to transplantation, we can speculate that transplant professionals will make choices that facilitates the greatest expected value (12). If an organ offer is declined, it is implied that the perceived costs (adverse outcomes) outweigh the benefits and we believe the recipient would be better off without accepting that particular organ offer.

**FIGURE 1:**
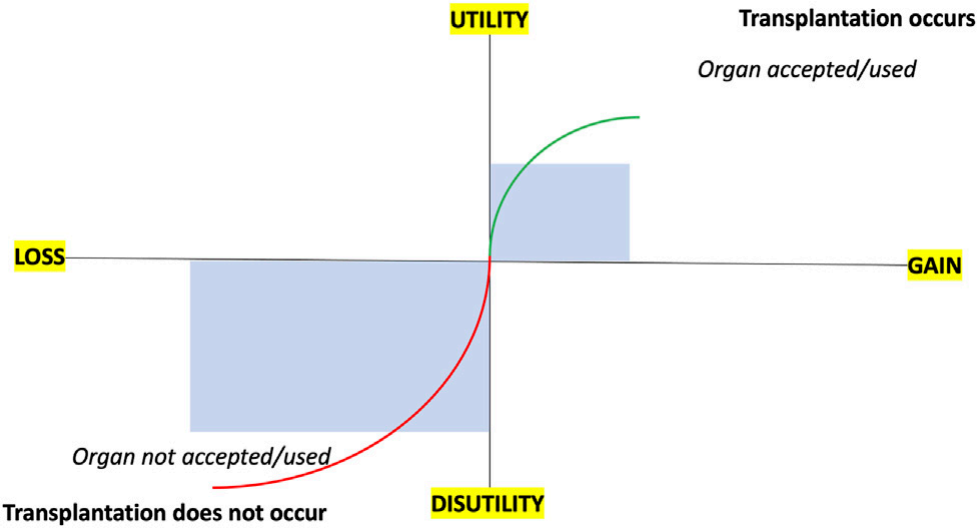
Schematic outlining prospect theory and loss aversion in relation to organ acceptance/utilization and transplantation.

However, it is not that simple or straightforward. Prospect theory, popularized by the Nobel Laureates Daniel Kahneman and Amos Tversky, would suggest individuals give more weight to factors framed as potential losses (risk) than to potential gains (benefits) (13). A transplant professional may overweigh the losses associated with accepting an organ and reject it even if the benefits outweigh the costs. This behavior is termed loss aversion and, when translated to transplantation, will manifest as usable organs being discarded (see Figure 1). A related behavioral factor that influences decision-making is risk aversion, where individuals choose a certain outcome over an outcome with less certainty. For transplant professionals, risk aversion will be the fear of larger loss (adverse outcome) resulting in settling for an unfavorable settlement (declining the kidney). Subjectively this attitude seems common, and we lack objective data about its true prevalence, but disparities in organ utilization data (either between (2, 4) or within centers) would support this assumption.

### Decision-Making Under Risk

The implanting surgeon is considered to be ultimately accountable for the use of a donated organ. However, while surgeons taking primary organ offers is the most common system, some centers and/or countries have physicians (14) or other transplant professionals as first contact. Many decisions are made outside working hours, often with limited information about the donor, working under significant stress and scrutiny. Time-pressured decision-making could introduce a perception that the penalty of accepting an organ may outweigh the penalty of declining the offer. Experiments undertaken in the setting of financial transactions show time-pressured decision-making has no effect on risk attitude for gains, but increased risk aversion for losses (15).

While shared decision-making with wait-listed candidates can attenuate some of this burden, this is challenging after hours or with time pressures to genuinely obtain informed consent. Shared decision-making with other members of the transplant professional team, either another surgeon or multi-disciplinary colleagues such as physicians or anesthetists, may absorb clinical responsibility across a wider team than the operating surgeon alone. However, the success or failure of this approach will be influenced by the overall risk appetite of the unit. Wider consultation may paradoxically lead to higher decline rates due to a dilution of clinical responsibility and a form of “*regression towards the mean*” (16).

### Clinical Decision-Making and Perception of Risk

Transplant professionals are willing to take risk to varying degrees, which is dependent upon their internal attitudes and perceptions (see Table 1) and external factors. While opinions will differ, and depend on personal bias, I suggest both extreme attitudes (risk avoidance and risk seeking) are undesirable for accepting organ offers, with risk tolerance the optimal “middle ground” with external factors all being equal.

**TABLE 1:** Spectrum of risk attitudes applied to transplantation.

Attribute	Risk avoiding	Risk averse	Risk neutral	Risk tolerant	Risk seeking
Focus	Focus mainly on negative risk and avoiding loss at all costs	Focus on managing or avoiding negative risk drives most decisions	Focus on managing risk balance between negative and positive	Focus is on positive risk, but negative risk is also considered	Focus on positive risk and maximising gain—*all-or-nothing* philosophy
Attitude	Risk is very bad and to be avoided at all costs	Risk is bad but acceptable in some circumstances	Risk is seen as both bad and good to be managed equally	Risk is good but unacceptable in some circumstances	Risk is very good and to be embraced at all costs
Transplant example	Declining all organ offers as ‘*no organ is ever risk-free*’	Declining most organ offers as ‘*no organ is risk-free*’	Accepting some organ offers but declining some as ‘*not every organ offer is better than no offer*’	Accepting most organ offers as ‘*any organ is better than no organ*’ in majority of cases	Accepting all organ offers as ‘*any organ is better than no organ*’
Risk versus benefit scale	Risk >>> Benefit	Risk > Benefit	Risk = Benefit	Risk < Benefit	Risk <<< Benefit
Optimal attitude^a^	Problematic	Questionable	Good	Ideal	Problematic

a Author opinion.

Risk perception varies among surgeons, and other transplant professionals, but has never been empirically studied. In the surgical literature, Dilaver et al. undertook a systematic review of surgeons’ perception of post-operative outcomes and risk (17). Twenty-seven studies comprising 20,898 patients undergoing a range of surgical procedures (but not solid organ transplantation) were included. Surgeons consistently overpredicted 30-day mortality rates and were outperformed by risk scoring tools in 6/7 studies comparing area under receiver operating characteristic curves (AUC). While surgeons’ prediction of general morbidity was good, being equivalent or better than risk prediction models, long-term outcomes were poorly predictive with AUC values ranging from 0.51 to 0.75.

There are limited data with regards to how surgical decision-making is linked to risk taking behavior (18). Sacks et al. conducted a randomized controlled trial exploring surgeons’ judgement and clinical decision-making to recommend surgery based upon four clinical vignettes (19). They were asked to assess risks (probability of serious complications or death) and benefits (recovery) of operative versus non-operative management and to rate their likelihood of recommending surgery. A national sample of surgeons were randomized into usage of clinical vignettes alone (control group; *n* = 384) versus supplementation by data from a risk calculator (risk calculator group; *n* = 395). The results demonstrated exposure to risk calculator data led to more homogenous and accurate judgments of operative risk among surgeons. However, while risk calculators may facilitate more informed discussions of various treatment options, they did not alter the likelihood of the surgeon recommending an operation on a 5-point scale (3.7 versus 3.7 per randomized arm, *p* = 0.76).

Given the same clinical scenarios in a different study with 767 participants, surgeons’ perceptions of treatment risks and benefits varied significantly and was highly predictive of their decision to operate (20). Analyzing hypothetical clinical vignettes, surgeons varied markedly in their assessment of the risks and benefits of operative and nonoperative management (range 4%–100%) and in their decision to operate (range 49%–85%). Surgeons were less likely to operate as their perception of operative risk increased and their perception of nonoperative benefit increased. By contrast, they were more likely to operate as their perception of operative benefit increased and their perception of nonoperative risk increased. Difference in risk/benefit perceptions explained 39% of the observed variation in decision to operate.

Some of this heterogeneity may be due to underlying personality traits of the operating surgeon. For example, Contessa et al. analyzed the association between personality factors (measured by the Myers-Briggs Type Indicator personality inventory), risk tolerance (measured by the Revised Physicians’ Reactions to Uncertainty) and Physician Risk Attitude scales in 27 surgeons at a single campus (21). From their analysis, surgeons with personality factors E (Extravert), T (Thinking), and P (Perception) demonstrated higher tolerance for risk, while surgeons with personality factors I (Introvert), F (Feeling), and J (Judgment) demonstrated risk aversion on the same measures. Factors such as gender, seniority and age may also play a role, with an increase in rationality and decrease in risk-readiness examples of profession-specific personality trait shifts (22).

### External Influences on Decision-Making Under Risk

Risk attitude will be influenced by external factors. Organ utilization will be sub-optimal if professionals accepting organ offers do not feel confident in the environment to perform surgery. Unfavorable environments lead to defensive medicine being practiced, even if contrary to evidence-based findings (23). In a cross-sectional survey of 220 physicians working in surgical specialities, defensive medicine was widely encountered with no correlation to age or experience (24). Transplantation occurs under regulatory oversight to ensure transplant centers achieve benchmark outcomes. However, pressure to achieve normative outcomes creates bias against accepting transplant risk (25). Center-specific factors weight heavily in decisions to accept organs. Their attenuation may alter risk perception, and improve organ acceptance/utilization in some cases, but will not totally overcome individual cognitive biases.

### Explaining Risk to the Wait-Listed Candidate

Wait-listed candidates also make decisions under stress, reliant upon good communication from the transplant professional for informed choice (26). Risk communication to patients about organ offers should incorporate discussion of risk, benefit and uncertainty that acknowledges the health literacy of the transplant candidate. However, risk communication to patients can be flawed. Objective evidence can be subjectively framed using different tricks to influence consent, with different examples of framing bias influencing decision-making (27). Therefore, even if organs are accepted, they may not be utilized after refusal by the wait-listed candidate during consent. While this may be appropriate in some cases, there will be scenarios where decision-making has been skewed towards risk aversion rather than risk tolerance by the inappropriate framing of risk by transplant professionals.

### Solutions: Targeting Behaviour Change for Improved Decision-Making

Before interventions can be developed, we must first define what optimal decision-making means. This can be subjective or heterogenous dependent upon individualized clinical scenarios. As described by Milkman et al., normative models provided by economic theorists offer a reasonable benchmark for how optimal decision-making is defined (28). According to these models, decision-making should be transitive, insensitive to minor changes in context, revealed preferences should be consistent with stated preferences, no systematic mathematical errors in judgment should arise, and a decision maker should remain satisfied after making a choice that their decision was correct after reflection. Most importantly, an optimal decision is one that a decision maker regards as the right choice regardless of whether they were evaluating their own decision or someone else’s.

To change decision-making behavior for organ offers, we must follow evidence-based methodology to firstly understand the underlying behavior and then utilize the correct intervention(s) for application. Systematic methods to understand behavior change exist, with a hierarchically-structured, taxonomy of 93 techniques used in behavior change therapy (BCT) clustered into 16 groups (29). Combining adequate assessment of the behavior to be changed (i.e., risk aversion), and application of the relevant theoretical constructs, a toolkit to design behavior change interventions to convert risk aversion to tolerance among transplant professionals is possible but requires investigation.

Other changes are required to reduce risk aversion. Transplant-specific guidelines that review decision-making barriers are required. These must provide evidence-based toolkits to support transplant professionals accepting organs and facilitation of risk communication. However, patient/public involvement is necessary to ensure communication is appropriately framed to aid understanding. Surgery must occur in adequately resourced and supported environments, with exact requirements varying between centers. This includes optimizing numbers of surgeons, physicians, allied health professionals, operating theatres, intensive care facilities, inpatient and outpatient follow-up facilities (16). This is unlikely to be achieved without significant monetary investment so other strategies (e.g., collaborative networks, shared decision-making, etc.) must be investigated for efficacy with quality improvement studies, audit and governance. Recognition that early post-transplant complications are not necessarily attributable to poor decision-making at the time of organ offer is important, for medico-legal purposes and regulatory oversight. This includes financial reimbursement, which may be insufficient with less-than-ideal organs that can lead to more complications and/or hospitalizations but are still in the best interest for patients.

Shared decision-making is important. This can be between patients and their healthcare providers, ensuring patients are at the centre of the transplantation decision (30). However, it is also desirable among the clinical transplant team, transforming individual professional risk to collective departmental risk. Responsibility must be shared with all multi-disciplinary professionals involved in the full spectrum from procurement to implantation. With adequate counselling, all parties must fully embrace the possibility of risk to gain the opportunity of benefit. This requires multi-stakeholder consensus, including patients and professionals, on optimized decision-making under risk for wait-listed candidates.

Fundamentally, we must learn to become risk tolerant. For example, early deaths after transplantation are usually rigorously investigated at a local level, with national involvement if centers deviate from the median. However, early post-transplant deaths are far outweighed by deaths for wait-listed candidates while awaiting a graft which have been hitherto ignored. As recently stated, “*we perceive greater risk in acts of commission than in acts of omission: if a patient dies during or after transplantation, it’s the doctor’s responsibility; if the patient dies from organ failure while awaiting a transplant, we can blame the indifference of the Universe*.” (31) Plus patient survival is not the only milestone to measure the success of transplantation. Quality of life benefits should also be considered in the decision-making of organ offers.

An adverse outcome is not necessarily an indicator that the decision to accept and/or transplant the organ was wrong. Indeed, I suggest any unit that has none is too risk averse and not transplanting enough. Paradoxically, higher surgical activity may lead to attenuation of adverse outcomes. Birkmeyer et al., in a study linking surgical skill and complication rates after bariatric surgery, observed technical skill was strongly correlated to procedural volume (32). Compared with the top quartile of skill, surgeons ranked in the bottom quartile experienced higher rates of reoperation, readmission within 30 days and return visits to the emergency department. Therefore, surgeons with low transplant activity will enter a Catch-22 situation; greater inclination for risk averse behavior that further reduces their procedural volume.

## Conclusion

Risk aversion by transplant professionals is an understandable but unwelcome barrier for optimized organ acceptance and/or utilization. Despite significant advancements in behavioral economics studying decision-making with risk, little reciprocal work has been undertaken in transplantation. National efforts to increase organ acceptance/utilization are important, with scientific and technological breakthroughs potentially ushering in exciting future possibilities (33, 34). However, we cannot overlook the human component to organ acceptance and/or utilization. While external factors are important, some center-specific and others regulatory or medico-legal, individual cognitive biases remain important. A concerted effort to study decision-making under risk for transplant professionals, and targeted behavioral measures to shift risk aversion to risk tolerance when accepting organ offers, should be strongly encouraged.

## Data Availability

The original contributions presented in the study are included in the article/Supplementary Material, further inquiries can be directed to the corresponding author.

## Abbreviations

BCT, behavior change therapy.
